# Prevention of Polyglycolic Acid-Induced Peritoneal Adhesions Using Alginate in a Rat Model

**DOI:** 10.1155/2015/403413

**Published:** 2015-05-21

**Authors:** Mari Matoba, Ayumi Hashimoto, Ayumi Tanzawa, Taichi Orikasa, Junki Ikeda, Yoshizumi Iwame, Yuki Ozamoto, Rie Abe, Hiroe Miyamoto, Chiko Yoshida, Toru Hashimoto, Hiroko Torii, Hideki Takamori, Shinichiro Morita, Hiroyuki Tsujimoto, Akeo Hagiwara

**Affiliations:** ^1^Medical Life System, Faculty of Life and Medical Science, Doshisha University, 1-3 Tatara Miyakodani, Kyotanabe, Kyoto 610-0394, Japan; ^2^Graduate School of Medicine for the Master's Course, Kyoto Prefectural University of Medicine, 465 Kajii-cho, Kawaramachi-Hirokoji, Kamigyo-ku, Kyoto 602-8566, Japan; ^3^Kusatsu General Hospital, 1660 Yabase-cho, Kusatsu, Shiga 525-8585, Japan; ^4^Department of Economics, Doshisha University, Imadegawa-Karasuma, Kamigyo-ku, Kyoto 602-8580, Japan; ^5^Biomedical Material Research Center, Doshisha University, 1-3 Tatara Miyakodani, Kyotanabe, Kyoto 610-0321, Japan

## Abstract

Postoperative intra-abdominal or intrathoracic adhesions sometimes cause significant morbidity. We have designed three types of alginate-based treatments using strongly cross-linked (SL), weakly cross-linked (WL), and non-cross-linked (NL) alginate with calcium gluconate. In rat experiments, we compared the antiadhesive effects of the three types of alginate-based treatments, fibrin glue treatment (a standard treatment), and no treatment against adhesions caused by polyglycolic acid (PGA) mesh (PGA-induced adhesions). The antiadhesive materials were set on the PGA sheet fixed on the parietal peritoneum of the abdomen. Fifty-six days later, the adhesions were evaluated macroscopically by the adhesion scores and microscopically by hematoxylin-eosin staining and immunostaining. We also tested the fibroblast growth on the surface of the antiadhesive materials *in vitro*. The antiadhesive effects of WL and NL were superior to the no treatment and fibrin glue treatment. A microscopic evaluation confirmed that the PGA sheet was covered by a peritoneal layer constructed of well-differentiated mesothelial cells, and the inflammation was most improved in the NL and WL. The fibroblast growth was inhibited most on the surfaces of the NL and WL. These results suggest that either the WL or NL treatments are suitable for preventing PGA-induced adhesions compared to SL or the conventional treatment.

## 1. Introduction

Intra-abdominal or intrathoracic adhesions develop after gastroenterological [1, 2], thoracic [3–6], and gynecological [7–9] surgeries. Adhesions sometimes cause bowel obstructions, chronic abdominal pain or discomfort, female infertility [2–4, 7, 8, 10, 11], and difficulties with subsequent surgeries, as well as prolonged hospitalization and hospital readmissions, which could have an impact on both the patient's well-being and healthcare costs [11, 12]. Therefore, postoperative adhesions should be prevented as much as possible.

A mesh of polyglycolic acid (PGA) is a widely used biomaterial during gastroenterological [13], thoracic [6, 14–16], and gynecological surgeries [9] and is used, for example, as reinforcement for weak tissues. The mild acidity of glycolic acid, produced during the nonenzymatic degradation of PGA [17], causes chronic inflammation, and adhesions subsequently occur around the site where the PGA mesh was placed [3, 18]. Thus PGA-induced adhesions have been problematic for a long time, but PGA continues to be used because it is a useful biomaterial.

In order to resolve the problems with PGA-induced adhesions, we utilized sodium alginate, which has previously been reported to prevent adhesions [19–21]. The present paper describes, for the first time, animal experiments in which two types of new antiadhesive materials comprising sodium alginate with and without a small amount of calcium gluconate solution had superior antiadhesive effects against PGA-induced adhesions to fibrin glue, the conventional antiadhesive material in the clinical setting [22, 23].

## 2. Materials and Methods

### 2.1. Materials

In this study we used a nonwoven PGA mesh (trade name: Neoveil, Gunze, Japan), fibrin glue (trade name: Beriplast P Combi-Set, CSL Behring, King of Prussia, PA, USA), sodium alginate powder (trade name: Alto, Kaigen, Japan) with a molecular weight ranging from 32,000 to 250,000, and calcium gluconate solution at a concentration of 8.5% (trade name: Calcicol, Nichi-Iko, Japan). The nonwoven PGA mesh was cut into square sheets 15 mm × 15 mm in size (PGA sheets) and sterilized with ethylene oxide for 22 hours, after which ethylene oxide gas was removed under conditions of decompression for one week. Fibrin glue was used according to the manual provided by the manufacturer in the form of a mixture of solutions A (fibrinogen and aprotinin) and B (thrombin and calcium chloride).

### 2.2. Placement of Alginate Gel on the PGA Sheet

Hirasaki et al. [19] reported that it takes a long time for powdered sodium alginate to dissolve in water and turn into a gel and that the powder form causes foreign body reactions, such as the induction of foreign body cells, when dispersed throughout the abdominal cavity. Therefore, the author recommended that alginate be administered in the form of a gel or freeze-dried flakes, which rapidly turn into a gel. Based on these recommendations, the alginate was administered as a gel in the present study.

### 2.3. Cross-Linking by Calcium Gluconate

We used three calcium ion conditions, specifically 1 mL (rich), 0.1 mL (low), and 0 mL (zero) of calcium solution. Condition 1 (rich): alginate is strongly cross-linked and turns into an insoluble hard gel [24, 25], which remains localized over the PGA sheet for a long time and hardly moves away. Therefore, it does not easily disperse throughout the abdominal cavity. Condition 2 (low): alginate is weakly cross-linked and forms a soft gel [26] that is gradually dissolved in water. The gel remains for a moderate amount of time then gradually moves away from the sheet, thus dispersing slowly throughout the abdominal cavity. Condition 3 (zero): alginate is not cross-linked and hence turns into a soluble gel [26, 27] that easily moves out from the sheet and is smoothly dispersed throughout the abdominal cavity. We subsequently tested these three types of alginate-based treatments: strongly cross-linked (SL), weakly cross-linked (WL), and non-cross-linked (NL).

### 2.4. Animal Protocol and Experimental Design

The present study comprised two parts, Experiments 1 and 2. In Experiment 1, 40 rats were randomly assigned to five experimental groups of eight rats each: a PGA alone group, fibrin group, SL group, WL group, and NL group. The antiadhesive effects of the three alginate-based treatments against PGA-induced adhesion were compared with those of PGA alone or fibrin glue treatment at a fixed alginate dose of 250 mg/rat. Upon obtaining the results of Experiment 1, we decreased the alginate dose in geometric progression (Experiment 2): 10, 20, 40, 80, or 160 mg/rat. Sixty rats were randomly assigned to 10 subgroups of six rats each, and the 10 subgroups were randomly divided into two major groups (WL and NL groups) composed of five subgroups. The five subgroups in the WL group (WL subgroups) included a 10 W subgroup, 20 W subgroup, 40 W subgroup, 80 W subgroup, and 160 W subgroup. Similarly, the five subgroups in the NL group (NL subgroups) included a 10 N subgroup, 20 N subgroup, 40 N subgroup, 80 N subgroup, and 160 N subgroup. The antiadhesive effects and accumulation of ascites fluid were evaluated.

The animal experiments were approved by the Doshisha University Animal Experimentation Committee. All surgical procedures and anesthesia protocols were conducted in accordance with the Animal Care Guidelines of Doshisha University. During the experimental period, all rats were housed separately and maintained under standard specific pathogen-free conditions (light-dark cycle: 12 : 12 hours, mean temperature: 23 degrees Celsius, and mean humidity: 50%). Standard laboratory rodent chow and water were available* ad libitum*. On the first day of the experiment, the health status of all rats was checked (diarrhea, unusual fur (loss or dirtiness), mucous discharge from the eyes or anus, and emaciation). Nonpregnant 8-week-old female Wistar/ST rats weighing 200 g were used for the analyses.

### 2.5. Surgical Technique

All operations were performed under sterile conditions and all procedures were performed by one surgeon. Rats inhaled diethyl ether (Wako Pure Chemical, Japan), and 5 mg sodium pentobarbital (trade name: Sommnopentyl, Kyoritsu Seiyaku, Japan) diluted in 1 mL of physiological saline solution was then administered intraperitoneally. Under general anesthesia, rats fixed in the dorsal position received a 5 cm long median transabdominal incision. A PGA sheet was fixed at the four corners on the visceral peritoneum of the right lateral abdomen with 7/0 polyvinylidene fluoride monofilament sutures for microsurgery (trade name: Asflex, Kono Seisakusyo, Japan)

#### 2.5.1. Experiment  1

After fixation, no antiadhesive materials were applied on the PGA sheets in the PGA alone group. Meanwhile, in the fibrin group, a mixture of solutions A and B at a concentration of 0.09 mL each (total: 0.18 mL), as an antiadhesive barrier, was sprayed over the fixed PGA sheet in order to entirely cover the surface of the sheet.

As for the three alginate groups before fixing the PGA sheet on the peritoneum, 0.1 mL of solution (1) was soaked up into the sheet and 125 mg of sodium alginate powder was subsequently sprinkled onto the sheet. The PGA sheet was fixed on the visceral peritoneum in the same manner as that used in the PGA alone group. Following fixation, 0.45 mL of solution (2) was sprayed over the PGA sheet and 125 mg of sodium alginate powder was sprinkled on the sheet. Finally, 0.45 mL of solution (3) was sprayed on the sheet. The details of the solutions (1–3) are summarized in Table 1.

Following the completion of these procedures, the laparotomy incision was closed using 4/0 polyamide sutures with two-layered sutures, the muscle layer was closed with continuous sutures, and the skin was closed with interrupted sutures. After surgery, all rats were maintained for 56 days under standard SPF conditions.

#### 2.5.2. Experiment  2

All operations were performed by the same surgeon who conducted the surgeries in Experiment 1. Before fixing the PGA sheet on the peritoneum, 0.1 mL of calcium solution (in the WL subgroups) or physiological saline solution (in the NL subgroups) was soaked up into the PGA sheet, and 10, 20, 40, 80, or 160 mg of sodium alginate powder was then sprinkled on the PGA sheet. The PGA sheet was subsequently fixed on the visceral peritoneum, and, after fixation, 0.9 mL of physiological saline solution was sprayed over the PGA sheet. Following the completion of these procedures, the laparotomy incision was closed in the same manner as in Experiment 1.

### 2.6. Macroscopic Evaluation of the Antiadhesive Effects

The macroscopic findings were evaluated by two examiners who were blinded to the rats' treatment. On postoperative day 56, when the PGA had been degraded and partly absorbed by the body [28], the rats were sacrificed by the intraperitoneal injection of a lethal dose of pentobarbital (3.5 mg/kg of body weight). After a full abdominal laparotomy, we observed the status of the adhesion on the PGA sheets macroscopically. We first recorded the extent and severity of adhesion according to an adhesion grading scale (adhesion score, Table 2 [29]) and subsequently recorded the number of adherent portions, namely, which intra-abdominal portions (tissues and organs) had adhered to the PGA sheet. The total number of adherent portions was compared between the treatment groups.

### 2.7. The Adherent Portions and the Correlations among the Adherent Portions, Adhesions Scores, and Alginate Doses

In Experiment 2, we investigated the correlations between (1) the alginate dose and total number of adherent portions, (2) the adhesion scores (extent and severity) and total number of adherent portions, and (3) the alginate dose and adhesion scores in the WL and NL subgroups.

### 2.8. Evaluation of Ascites

We examined the correlation between the volume of ascites and the antiadhesive effects in Experiments 1 and 2. For each of the 90 rats in the WL and NL groups, the volume of ascites was classified into four classes using the ascites score shown in Table 3. A dot was placed on each rat at the point shown by the *x*- and *y*-axes.

### 2.9. Microscopic Study of Hematoxylin-Eosin (HE) Staining

All rats were subjected to a microscopic study. The peritoneal wall where the PGA sheet had been fixed was surgically removed* en bloc* as a specimen for microscopic examination. Specimens were fixed in 10% formalin solution and were prepared as thin (3 *μ*m in thickness) sections stained with a hematoxylin-eosin (HE) using standard procedures for histological examinations. HE sections were reviewed for all rats.

### 2.10. Immunohistochemical Study

We selected one section from all five groups in Experiment 1 for the immunohistochemical analysis. As a primary antibody, we used an anti-human mesothelial cell antibody (HBME-1, Serotec, Japan), which is available for the staining of mesothelial cells [30].

In order to investigate whether or not macrophages were inflammatory or tissue-remodeling, we used an anti-rat CD68 mouse monoclonal antibody (Clone ED1, Serotec), anti-rat CD163 mouse monoclonal antibody (clone ED2, Serotec), and anti-rat CD86 mouse monoclonal antibody as the primary antibodies, which could differentiate inflammatory macrophages (M1 macrophages) [31], tissue-remodeling macrophages (M2a and M2c) [31, 32], and both macrophages (M1 and M2b) [32], respectively. All sections were cut to 3 *μ*m in thickness and were deparaffinized and hydrophilized. Antigen activation was performed by immersing the samples in a warm bath with EDTA at pH 9 (20 minutes at 95°C) (HBME-1), with heat and pressure treatment with 10 mM sodium citrate buffer solution at pH 6.0 (15 minutes at 121°C) (CD68), which were not treated (CD163) or were treated with proteinase K (5 minutes at room temperature) (CD86), respectively. The sections were washed with distilled water and successively treated with 3% H_2_O_2_ (10 minutes at room temperature). The sections were then washed with distilled water and successively rinsed in 50 mM Tris-HCl buffer, pH 7.6, containing 0.05% tween-20 and 0.15 M NaCl (TBST). The sections were incubated overnight at 4°C (HBME-1 (1 : 50 dilution), CD68 (1 : 1000), CD163 (1 : 50), and CD86 (1 : 50), all of which were diluted with ChemMate (Dako, Japan) and were successively rinsed in TBST. After that, the sections for HBME-1 and CD68 were incubated with the Envision+ polymer reagent (Dako, Japan). For the CD163 and CD86 staining, the sections were subsequently incubated with a Simple Stain rat MAX-PO (MULTI) kit (Nichirei, Japan), each for 30 minutes at room temperature. After being rinsed in TBST, all sections were incubated with a 3,3′-diaminobenzidine tetrahydrochloride (DAB+) substrate kit (Dako). After washing, all sections were subjected to counterstaining, dehydration, penetration, and mounting.

### 2.11. The Fibroblast Growth on the Antiadhesive Materials* In Vitro*

Cultured rat fibroblasts, which were established from the subcutaneous tissue under the back skin of a healthy Wistar S/T rat (seven weeks old, 200 g), were retrieved in the form of a single cell suspension with D-MEM medium (Wako Pure Chemical) containing 10% bovine serum. The cell suspension was diluted with the same medium to 1.5 × 10^4^ cells/mL. This suspension was poured at 100 *μ*L/well into the coated wells in the plates described above so that there were 1.0 × 10^4^ human cells/well (*n* = 4) as a control. The cell suspension was also poured into a humidified incubator with 5% CO_2_ at 37°C.

For the cell culture, we used 24-well culture plates with wells 15 mm in diameter without a coating (Becton, Dickinson and Company Ltd., Franklin Lakes, USA). The 24 wells were divided into three groups: the SL group, WL group, and NL group. Fifty mg of sodium alginate and the calcium gluconate and/or physiological saline solutions were combined in the same proportions by weight. The type and volume of solution are summarized in Table 4. One, three, five, and seven days after seeding, the viable cell number in each well was counted with the ATP assay using an ATP Lite Kit (Perkin Elmer, Waltham, USA). For each time point, four wells for each experimental group were examined.

### 2.12. Statistical Analysis

The statistical analyses were performed using the software program, “StatMate.” The adhesion scores (extent and severity) were assessed using the Kruskal-Wallis and Mann-Whitney *U* tests, and the statistical significance of differences in the total number of adherent portions and the correlations between groups was assessed using Pearson's chi-square test (*χ* ^2^). A regression analysis was employed to determine the correlation between the antiadhesive effects and the volume of ascites. Cell growth was analyzed using a one-way analysis of variance (ANOVA) and the Tukey test was used as a post hoc test. A value of *P* < 0.05 was considered to be statistically significant.

## 3. Results

### 3.1. Macroscopic Findings of the Antiadhesive Effects

**Figure 1:**
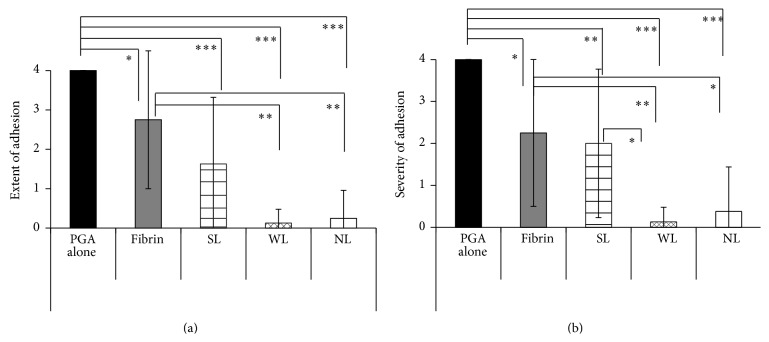
Adhesion scores in Experiment 1. (a) Extent and (b) severity of adhesion. The columns indicate the mean scores and the bars indicate the standard deviation. The black column shows the scores in the PGA alone group, the gray column shows the scores in the fibrin group, the horizontally striped column shows the scores in the SL group, the mesh pattern column shows the scores in the WL group, and the white column shows the scores in the NL group. *P* values < 0.05 are marked by an asterisk (*∗*), those <0.01 are marked by double asterisks (*∗∗*), and those <0.001 are marked by triple asterisks (*∗∗∗*).

**Figure 2:**
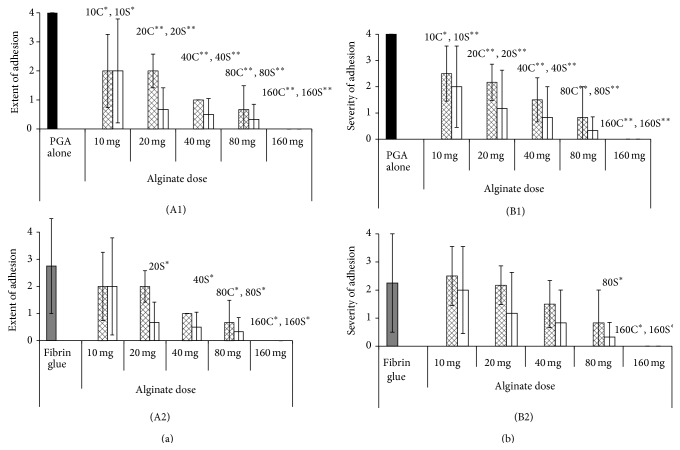
Adhesion scores in Experiment 2. The columns show the mean scores and bars indicate the standard deviation. The black column shows the scores in the PGA alone group, the gray column shows the scores in the fibrin group, the mesh pattern columns show the scores in the five WL subgroups, and the white columns show the scores in the five NL subgroups. The extent of adhesion is described in A1 and A2, and the severity of adhesions is described in B1 and B2. The adhesion scores in the WL and NL groups were compared with those in the PGA alone group (A1 and B1) and the fibrin group (A2 and B2). *P* values < 0.05 are marked by an asterisk (*∗*) and those <0.01 are marked by double asterisks (*∗∗*) and shown in the upper right of the graph in capital letters.

The adhesion scores (extent and severity) are expressed as the mean ± standard deviation and summarized in Table 5. Figures 1(a) (extent) and 1(b) (severity) show the scores for Experiment 1 and Figures 2 (A1 and A2 for extent) and 2 (B1 and B2 for severity) show the scores for Experiment 2.

#### 3.1.1. Experiment  1

Regarding the extent of adhesion, the scores in the WL and NL groups were significantly different from those in the PGA alone group (*P* < 0.001) and the fibrin group (*P* < 0.01). In addition, the scores in the SL and fibrin groups were significantly different from those in the PGA alone group (*P* < 0.001–0.05). As to the severity of adhesion, the scores in the WL and NL groups were significantly different from those in the PGA alone group (*P* < 0.001) and the fibrin group (*P* < 0.01–0.05), the scores in the WL group were significantly different from those in the SL group (*P* < 0.05), and the scores in the SL and fibrin groups were significantly different from those in the PGA alone group (*P* < 0.01–0.05).

#### 3.1.2. Experiment  2

The adhesion scores decreased as the alginate dose increased in the WL and NL subgroups. With respect to the extent of adhesion, all scores in the WL and NL subgroups were significantly smaller than those in the PGA alone group (*P* < 0.01–0.05), and the scores in the 80 W, 160 W, 20 N, 40 N, 80 N, and 160 N subgroups were significantly smaller (*P* < 0.05) than those in the fibrin group. Regarding the severity of adhesion, the scores in the WL and NL subgroups were significantly smaller than those in the PGA alone group (*P* < 0.01–0.05), and the scores in the 160 W, 80 N, and 160 N subgroups were significantly smaller (*P* < 0.05) than those in the fibrin group.

### 3.2. Adherent Portions and the Correlations

Table 6 shows the details of the adherent portions. The adherent portions included the omentum, gonadal fat, mesenterium, and part of the intestines in Experiment 1 and the omentum and gonadal fat in Experiment 2.

In Experiment 1, the total number of adherent portions was significantly different among the five groups (*P* < 0.001), and that observed in the WL and NL groups was significantly different from that noted in the PGA alone group (*P* < 0.001) and fibrin group (*P* < 0.01). In Experiment 2, the total number of adherent portions decreased as the alginate dose increased in both the WL and NL subgroups. In addition, the total number of adherent portions differed significantly (*P* < 0.001) among the WL subgroups, while that in the NL subgroups did not. Individually, the total number in the 160 W subgroup was significantly different from that observed in the 10 W, 20 W, and 40 W subgroups and the number in the 80 W subgroup was different from that seen in the 10 W subgroup (*P* < 0.01–0.05).

**Figure 3:**
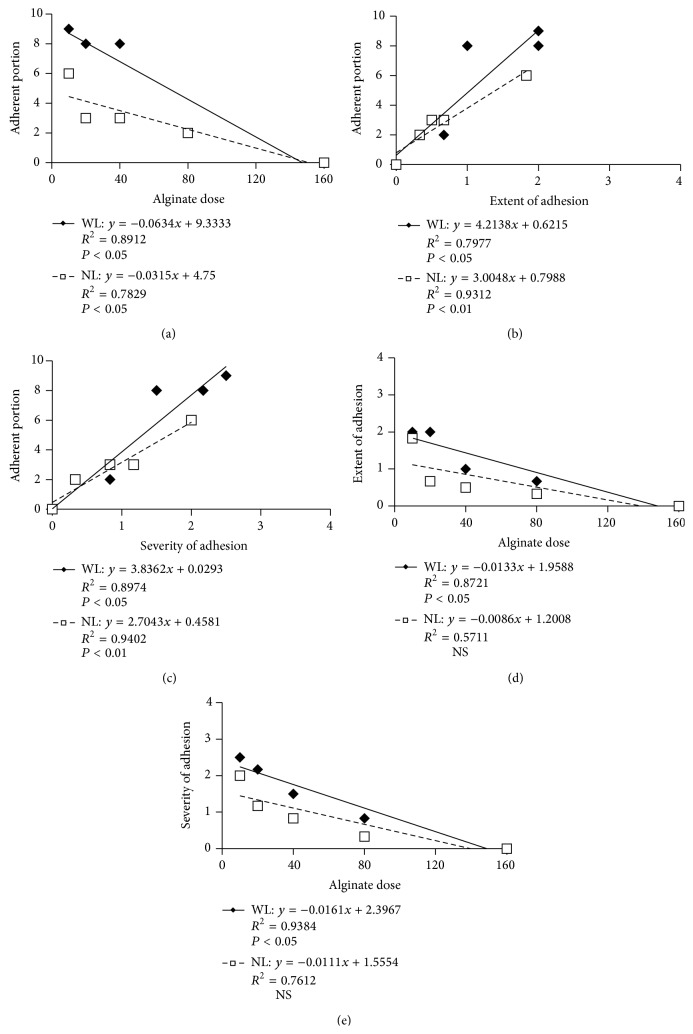
Correlations between the alginate dose, total number of adherent portions, and adhesion scores (extent and severity). The diamonds and squares indicate the WL and NL subgroups, respectively.

Figures 3(a) to 3(e) show the correlations between the alginate dose and total number of adherent portions (Figure 3(a)), the adhesion scores and total number of adherent portions (Figure 3(b) for the extent and Figure 3(c) for the severity), and the alginate dose and adhesion scores (Figure 3(d) for the extent and Figure 3(e) for the severity). Except for the correlations between the alginate dose and adhesion scores (extent and severity) in the NL subgroups, the correlation coefficients were all significant (*P* < 0.01–0.05).

### 3.3. Evaluation of Ascites

**Figure 4:**
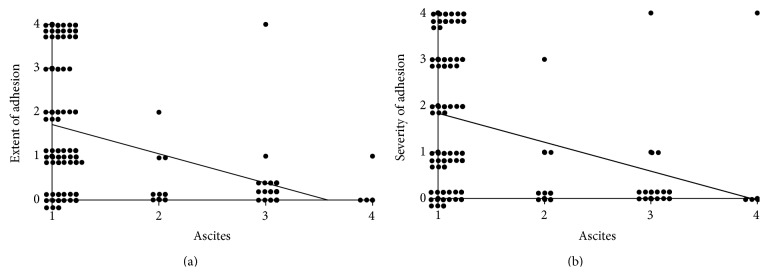
Correlations between the antiadhesive effects and the volume of ascites. (a) The correlation is expressed as the equation *Y* = −0.665*X* + 2.382 (*R* ^2^ = 0.1655, *P* < 0.001). (b) The correlation is expressed as the equation *Y* = −0.6257*X* + 2.4663 (*R* ^2^ = 0.1356, *P* < 0.001).

The ascites fluid was examined in the WL and NL groups only. Figures 4(a) and 4(b) show the correlations between the ascites score and adhesion scores (Figure 4(a) for extent and Figure 4(b) for severity). Consequently, the volume of ascites exhibited a strong inverse correlation with both the extent and severity of adhesion.

### 3.4. Microscopic Study of Hematoxylin-Eosin (HE) Stained Sections

**Figure 5:**
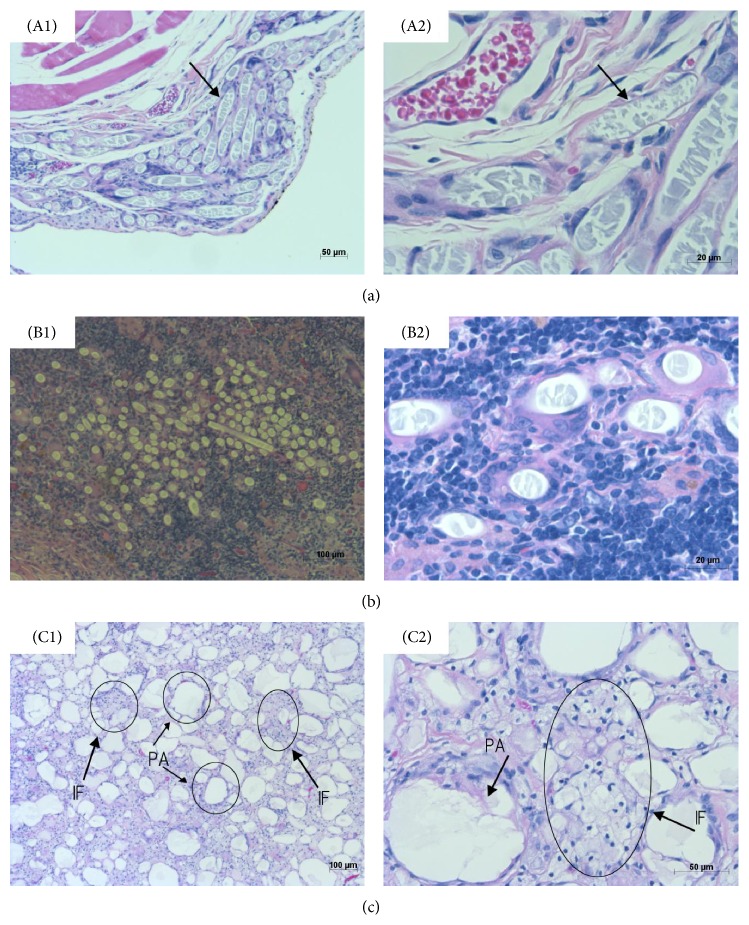
Microscopic findings of HE staining. A1 and A2 show the common changes in the five groups. The PGA fibers decreased in size. The PGA fibers turned into flakes (arrow), with each PGA fiber covered by macrophages and collagen-like materials. B1 and B2 show the specific changes in the fibrin group. Inflammatory cells, mainly lymphocytes, had accumulated. Huge lymph follicles were found around the PGA fibers (3/8 rats). C1 and C2 show the specific changes in the three alginate groups. There were two forms of residual alginate: island formation (IF) due to the accumulation of many macrophages ingesting alginate and a “pool” of alginate (PA) in a free state. The scale bars are 50 *μ*m (A1), 20 *μ*m (A2), 100 *μ*m (B1), 50 *μ*m (B2), 100 *μ*m (C1), and 50 *μ*m (C2).

The microscopic findings of the HE sections from Experiment 1 are summarized in Tables 7(a) and 7(b), and six photomicrographs are shown in Figures 5(A1) to 5(C3).  Table 7(a) shows the common changes in the five groups and specific changes in the fibrin and three alginate groups. Common changes had three steps: the first step was that the PGA fibers decreased in size, the second step was that the PGA fibers turned into flakes, and the third step was that each PGA fiber was covered by macrophages and collagen-like substrates. Thus, a complex was formed by the fibers, macrophages, and collagen-like substrates. The specific changes in the fibrin group had two steps: the first step was that a lot of inflammatory cells, mainly lymphocytes, accumulated all over the microscopic visual fields (8/8 rats) and the second step was that huge lymph follicles were found (3/8 rats). This accumulation of lymphocytes was remarkable compared with that in the other groups. The specific change in the three alginate groups was residual alginate form: one form was island formation by the gathering of many macrophages ingesting alginate (IF) and the other form was a “pool” of alginate in a free state (PA). Many of the “pools” were scattered around the PGA fibers.  Table 7(b) shows the amounts of IF and PA. The changes in lymphocyte accumulation, fibroblast infiltration, and fibrosis were weak in the three alginate groups, whereas these findings were remarkable in the other two groups. The microscopic findings in Experiment 2 were principally similar to those observed in the WL and NL groups in Experiment 1. The degree of lymphocyte infiltration, fibroblast growth, and collagen fiber accumulation was weak, although ascites accumulation was detected.

### 3.5. Immunohistochemical Study

**Figure 6:**
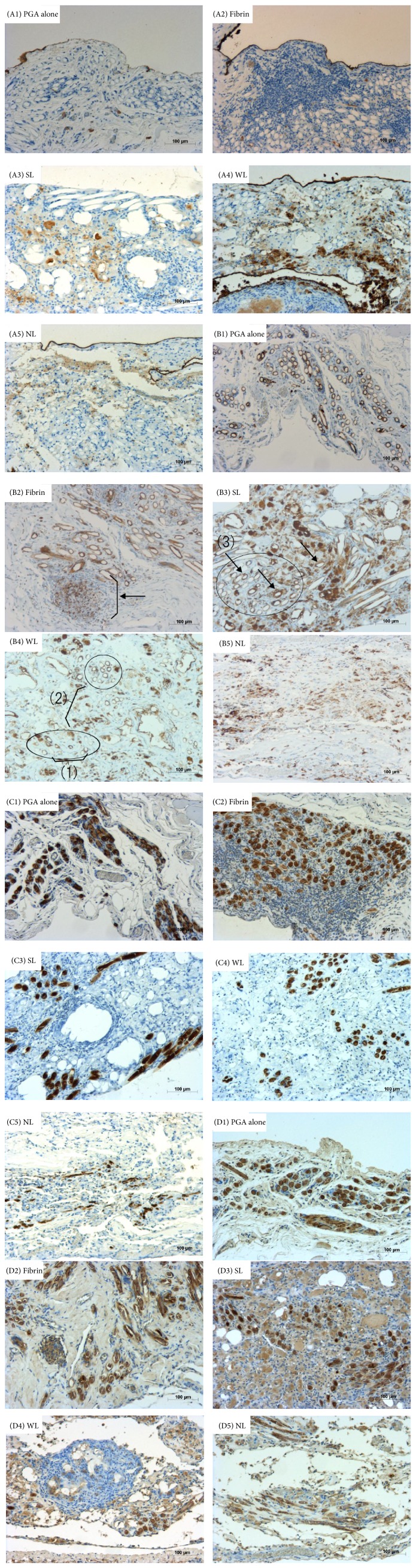
Immunohistochemical staining with HBME-1, CD68, CD163, and CD86. A1 to A5 show the results of HE staining, B1 to B5 show CD68 staining, C1 to C5 show CD163 staining, and D1 to D5 show CD86 staining. (B2) CD68-positive cells were found at sites of accumulation of lymphocytes (arrow). (B3 and B4) The circles show PGA bundles and (1)–(3) show positive cells: (1) cells between the fibers in the PGA bundles, (2) cells in the space between the PGA bundles, and (3) cells at the surface of the PGA fiber. All scale bars are 100 *μ*m.

Table 8(a) summarizes immunohistochemical features and Figures 6 (A1 to A5) represent the microscopic views of HBME-1 staining. HBME-1 was most clearly stained in the single cell layer covering the surface of the tissue over the PGA sheet in the NL group, followed by the fibrin group and the WL group, and the staining was unclear in the PGA alone group and the SL group. The HBME-1 staining was poor on the surface over the lymph follicles in the fibrin group and over the PGA fibers in the PGA alone group.

Figures 6 (B1 to B5), 6 (C1 to C5), and 6 (D1 to D5) show the typical views of CD68, CD163, and CD86 staining, respectively. The PGA sheet had two different fiber structures: one was a fiber bundle where the PGA fibers were dense; the other was space between the bundles. Macrophages were divided in three groups according to their locations: (1) macrophages between the PGA fibers in the PGA bundles, (2) macrophages in the space between the bundles, and (3) macrophages on the surface of the PGA fibers. Table 8(b) represents the immunohistochemical features using CD68, CD163, and CD86 staining for the macrophage phenotypes (inflammatory or tissue-remodeling). (1) The macrophages in the space between the bundles showed CD163-positive cells most predominantly between the PGA fibers in the NL group, whereas, in the SL group, CD163-positive cells were hardly detected. (2) With regard to the macrophages in the space between the bundles, CD68-positive cells and CD86-positive cells were predominant in the SL group, but not in the NL group. (3) As for the macrophages on the surface of the PGA fiber, there were no differences among the groups.

### 3.6. Fibroblast Growth on the Antiadhesive Materials* In Vitro*

**Figure 7:**
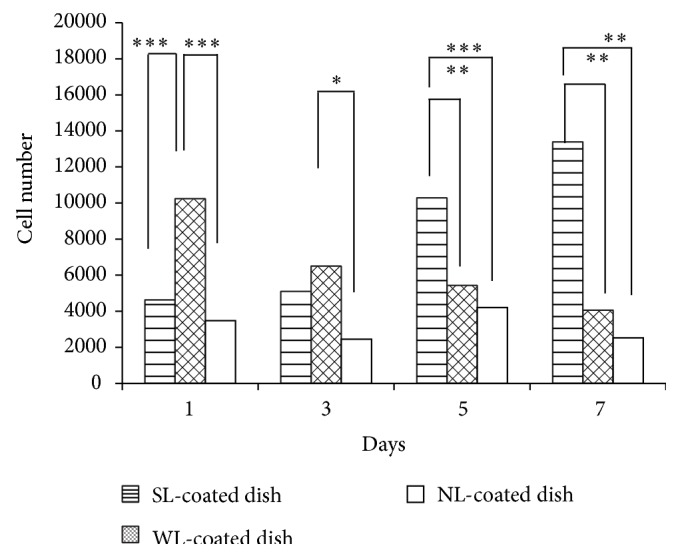
Fibroblast growth on the antiadhesive materials* in vitro. *The columns indicate the mean cell number and the bars indicate the standard deviation. The horizontally striped columns, mesh pattern columns, and white columns indicate the scores in the SL group, WL group, and NL group, respectively. *P* values < 0.05 are marked by an asterisk (*∗*), those <0.01 are marked by double asterisks (*∗∗*), and those <0.001 are marked by triple asterisks (*∗∗∗*).

As shown in Figure 7, the fibroblasts in the three alginate groups did not grow on all alginate gels. The fibroblasts hardly attached to the alginate surface, and the final cell growth on day 7 was significantly lower in the NL and WL groups than in the SL group (*P* < 0.01).

## 4. Discussion

First, as the alginate dose was high, we compared the adhesion scores of the three alginate-based treatments (SL, WL, and NL alginate) with those of no treatment (PGA alone) as well as conventional treatment (fibrin glue). The results showed that high doses of the WL and NL treatments had superior antiadhesive effects to both PGA alone and fibrin glue. Subsequently, as the WL and NL alginate doses were low, we investigated (1) whether a lower alginate dose provides significant antiadhesive effects against PGA-induced adhesion and (2) which alginate doses show antiadhesive effects corresponding to those of fibrin glue. The results showed that (1) both WL and NL treatment effectively prevented PGA-induced adhesion, even at the lowest alginate dose, while (2) 80 mg (NL) and 160 mg (WL and NL) of alginate displayed superior antiadhesive effect to fibrin glue. In brief, the alginates, even at very low doses, exerted strong antiadhesive effects against PGA-induced adhesion under low- and zero-calcium conditions.

We did not consider the ascites caused by the alginate to be inflammatory based on the size of the alginate molecules and the microscopic findings of inflammation, including inflammatory cell infiltration. Our alginate has a high molecular weight, and such molecules are large enough to prevent the material from passing through the endothelium of blood capillaries under the peritoneum in the peritoneal cavity [33, 34]. Therefore, large molecules are absorbed very gradually into the lymphatic system [34]. The long-term existence of large molecules results in the accumulation of water in the peritoneal cavity. According to the microscopic findings, the degree of inflammation was relatively low in the rats with ascites versus those without. Aksoy et al. [35] showed that the existence of liquid inside the abdomen, or ascites, has a preventive effect against postoperative adhesion caused by peritoneal injury in a rat experiment. Moreover, the volume of ascites strongly correlates with the antiadhesive effects. Hence, the ascites detected in our experiments may have contributed to the observed antiadhesive effects.

In order to detect well-differentiated mesothelial cells, HBME-1 is usually used as a mesothelial cell marker to recognize the microvillus structure of mesothelial cells [30]. After intraperitoneal injury or inflammation, an inflammatory stimulus leads to detaching mesothelial cells from the basement membrane and changes their morphology and function [36, 37]. Then, the injured surface is repaired in the tissue repair and remodeling phase. Finally, when adhesion does not occur the injured surface is covered by a repaired peritoneal layer of mesothelial cells. These well-differentiated mesothelial cells are characterized by a microvillus structure [38]. Therefore, the layer of HBME-1-positive cells indicates that there is a peritoneal layer constructed of mesothelial cells that are structurally and functionally well-differentiated. After the peritoneum has been repaired over the injured surface, the injured surface does not adhere. On postoperative day 56, the layer of mesothelial cells was formed clearly on the soft gels well over the PGA sheet (WL and NL groups) but was poorly formed at the free surface of lymph follicles (fibrin group) and at the free surface of residual alginate (SL group). Thus WL and NL treatments showed good antiadhesive results microscopically, while the SL and fibrin glue treatments showed lesser effects.

Macrophages have recently been divided into two phenotypes: M1 macrophages, which are inflammatory, and M2 macrophages, which are tissue-remodeling [31, 32]. M2 macrophages include at least three subsets (M2a, M2b, and M2c). The M2a and M2c macrophages are involved in tissue-remodeling, while M2b macrophages are inflammatory. M1 and M2b macrophages are characterized by the expression of CD68 and CD86 on their surface, respectively. On the other hand, M2a and M2c macrophages are characterized by the expression of CD163. As the calcium ion concentration decreased, more tissue-remodeling macrophages were found, whereas fewer inflammatory macrophages were detected. Thus, analyses of the macrophage phenotype and subsets showed that NL treatment had the best “tissue-remodeling” profile and SL treatment had the most inflammatory profile of the three types of alginate-based treatments.

We examined the extent of fibroblast growth on the antiadhesive materials* in vitro* because fibroblasts induce adhesion. Consequently, the* in vitro* examinations revealed that the fibroblasts hardly adhered to or proliferated on the surface of the soft and soluble gel (WL and NL), while they grew weakly on the hard gel (SL). The above-mentioned properties of the WL and NL treatments likely contributed to their superior antiadhesive effects compared to the SL alginate and fibrin glue. Doyle et al. [39] isolated human dermal fibroblasts and other cells and investigated the proliferation of these cells in a calcium alginate suspension. The authors subsequently reported that the fibroblasts grew more successfully on the calcium alginate than on the sodium alginate.

In summary, the NL group exhibited the smallest adhesion scores, even when the alginate dose was decreased, with few fibroblasts or inflammatory macrophages on the NL alginate. Moreover, microscopic examinations revealed many tissue-remodeling macrophages and well-differentiated mesothelial cells covering the surface of the PGA sheet. These results show that sodium alginate has the best antiadhesive effect of all of the tested antiadhesive materials.

The present paper focused on sodium alginate as an antiadhesive material, substituting it for fibrin glue, which has well-known antiadhesive effects and is used in the clinical setting [22, 23], as well as in animal experiments [40–45]. Some previous reports have shown that alginate has good antiadhesive effects [20, 21] and great bioabsorbable properties and biocompatibility [24–27, 39, 46–48]. In addition, the risk of infection by alginate has been known to be much lower than that by fibrin glue [27, 49], because alginate is extracted from seaweed. Fibrin glue is derived from human blood and thus is associated with a clinical risk of pathogenic infections [48]. For example, human parvovirus B19 cannot be removed from plasma-derived products even by pasteurization at 60°C for 10 hours [48].

It should be noted that our sodium alginate (trade name: Alto) is a clinical hemostatic agent, while the calcium gluconate solution (trade name: Calcicol) is a clinical injection used to supplement calcium. We selected only clinically available drugs as alginate-based treatments because surgeons can prepare this new antiadhesive material at their discretion as needed during surgery, and extensive studies of the safety of these drugs have already been performed.

Our intra-abdominal adhesion model was similar to the experimental rat model established by Junge et al. [50]. We assessed the antiadhesive effects on postoperative day 56, when the PGA mesh would be degraded into fine fragments and the tensile strength of the PGA mesh would be expected to be 0 [28]. At that point in time, the postsurgical inflammatory reaction had subsided [51]. We thought that this time point would provide a good assessment of the antiadhesive effects of the treatments, although the antiadhesive effects of alginate have generally been evaluated at earlier time points [50, 52].

## 5. Conclusion

The present study showed that two types of newly developed antiadhesive materials composed of sodium alginate alone and weakly cross-linked alginate with calcium, which can be prepared from clinically available drugs, effectively prevented PGA-induced adhesions. These results suggest that the new antiadhesive materials would be clinically useful to prevent the adhesions induced by PGA sheets, which are widely used in many clinical fields.

## Figures and Tables

**Table 1:** Three alginate groups in Experiment 1.

Experimental groups	Solutions		
	(1)	(2)	(3)
SL	Calcium gluconate	Calcium gluconate	Calcium gluconate
WL	Calcium gluconate	Physiological saline	Physiological saline
NL	Physiological saline	Physiological saline	Physiological saline

**Table 2:** Adhesion score.

Category and description	Score
Extent	
No involvement	0
≤25% of the site involved	1
≤50% of the site involved	2
≤75% of the site involved	3
≤100% of the site involved	4
Severity	
No adhesion present	0
Adhesions fall apart	1
Adhesions can be lysed with traction	2
Adhesions requiring <50% sharp dissection	3
Adhesions requiring >50% sharp dissection	4

**Table 3:** Ascites score.

Macroscopic accumulation of the ascites	Score
No accumulation	1
Accumulation limited in one side (right or left) gutter of the abdomen	2
Accumulation limited in bilateral gutters of the abdomen	3
Accumulation over the bilateral gutters	4

**Table 4:** Type and volume of solution(s) sprayed after sprinkling 50 mg of alginate.

Experimental groups	Type of solution	Volume of solution
SL	0.2 mL	Calcium solution
WL	(1) 0.02 mL + (2) 0.18 mL	(1) Calcium solution + (2) physiological saline solution
NL	0.2 mL	Physiological saline solution

**Table 5:** Adhesion scores (extent and severity).

Experiment number	Experimental groups	Mean ± SD	
		Extent of adhesion	Severity of adhesion
1	PGA alone group	4.0 ± 0	4.0 ± 0
	Fibrin group	2.8 ± 1.8	2.3 ± 1.8
	SL group	1.6 ± 1.7	2.0 ± 1.8
	WL group	0.1 ± 0.4	0.1 ± 0.4
	NL group	0.3 ± 0.7	0.3 ± 0.7

2	10 W subgroup	2.0 ± 1.3	2.5 ± 1.1
	20 W subgroup	2.0 ± 0.6	2.2 ± 0.7
	40 W subgroup	1.0 ± 0	1.5 ± 1.0
	80 W subgroup	0.7 ± 0.8	0.8 ± 1.2
	160 W subgroup	0 ± 0	0 ± 0
	10 N subgroup	1.8 ± 1.5	2.0 ± 1.6
	20 N subgroup	0.7 ± 0.8	1.2 ± 1.2
	40 N subgroup	0.5 ± 0.6	0.8 ± 1.0
	80 N subgroup	0.3 ± 0.5	0.3 ± 0.5
	160 N subgroup	0 ± 0	0 ± 0

The adhesion scores (extent and severity) are expressed as the mean ± standard deviation.

**Table 6:** Number of adherent portions where the PGA sheet adhered.

Experiment number	Experimental groups	Omentum	Gonadal fat	Intestine (part)	Mesenterium	Total
1	PGA group	8/8	5/8	1/8	0/8	14/32
	Fibrin group	6/8	4/8	0/8	0/8	10/32
	SL group	4/8	1/8	1/8	1/8	7/32
	WL group	1/8	0/8	0/8	0/8	1/32
	NL group	1/8	0/8	0/8	0/8	1/32

2	10 W subgroup	4/6	5/6	0/6	0/6	9/24
	20 W subgroup	2/6	6/6	0/6	0/6	8/24
	40 W subgroup	5/6	3/6	0/6	0/6	8/24
	80 W subgroup	1/6	1/6	0/6	0/6	2/24
	160 W subgroup	0/6	0/6	0/6	0/6	0/24
	10 N subgroup	4/6	2/6	0/6	0/6	6/24
	20 N subgroup	2/6	1/6	0/6	0/6	3/24
	40 N subgroup	2/6	1/6	0/6	0/6	3/24
	80 N subgroup	2/6	0/6	0/6	0/6	2/24
	160 N subgroup	0/6	0/6	0/6	0/6	0/24

**(a):** 

Common changes in all groups	

PGA fibers decreased in size	
PGA fibers turned into flakes	
Each PGA fiber was covered by macrophages and collagen-like substrates	

Specific changes in each group	
Fibrin group	The three alginate groups

Inflammatory cells, mainly lymphocytes, accumulated (8/8 rats)	Residual alginate was seen in two forms
	(1) Island-formation by gathering of many macrophages ingesting alginate (IF)
Huge lymph follicles were found around PGA fibers (3/8 rats)	(2) “Pool” of alginate in the free state (PA)

Common changes included three steps in the five groups. Specific changes in the fibrin group included two steps and those in the three alginate groups included residual alginate in two different forms (IF and PA).

**(b):** 

Experimental groups	IF	PA
SL group	Small amount	Large amount
WL group	Small amount	Moderate amount
NL group	Small amount	Small amount

IF: many macrophages were gathered in the form of an island, where they ingested the alginate.

PA: there was a “pool” of alginate in a free state.

**(a):** 

Experimental groups	The single cell layer stained with HBME-1
PGA alone	+
Fibrin	++
SL	+
WL	++
NL	+++

HBME-1 stained the single cell layer covering the tissue surface facing the peritoneal cavity. The HBME-1 staining was scored as follows: +++: clearly stained; ++: stained; and +: unclear. The HBME-1 staining was poor on the surface over the lymph follicles in the fibrin group and over the PGA fibers in the PGA alone group.

**(b):** 

Experimental groups	(1)			(2)			(3)		
	CD68	CD86	CD163	CD68	CD86	CD163	CD68	CD86	CD163
PGA alone	±	±	○	±	±	×	○	○	○
Fibrin	+	±	○	+	±	×	○	○	○
SL	+++	+++	×	+++	+++	×	○	○	○
WL	++	++	○	++	++	×	○	○	○
NL	+	+	*⦾*	+	+	×	○	○	○

The macrophages were stained for CD68, CD86, and CD163 in three locations (1–3). The staining was described as follows: *⦾*: mostly positive cells; ○: some positive cells; and ×: no positive cells. The visually recognized positive cells were scored as follows: +++: high; ++: medium; +: small; ±: few.

(1) Cells between the fibers in the PGA bundles.

(2) Cells in the space between the PGA bundles.

(3) Cells on the surface of the PGA fiber.
